# Small Molecule Binds with Lymphocyte Antigen 6K to Induce Cancer Cell Death

**DOI:** 10.3390/cancers12020509

**Published:** 2020-02-22

**Authors:** Senyi Benti, Purushottam B. Tiwari, Dustin W. Goodlett, Leily Daneshian, Grant B. Kern, Mark D. Smith, Aykut Uren, Maksymilian Chruszcz, Linda S. Shimizu, Geeta Upadhyay

**Affiliations:** 1 Department of Oncology, Georgetown University Medical Center, Washington, DC 20057, USA; ligaba@carilionclinic.org (S.B.); pbt7@georgetown.edu (P.B.T.); au26@georgetown.edu (A.U.); 2 Department of Chemistry and Biochemistry, University of South Carolina, Columbia, SC 29208, USA; dustinwg@email.sc.edu (D.W.G.); leily@email.sc.edu (L.D.); MDSMITH3@mailbox.sc.edu (M.D.S.); Chruszcz@mailbox.sc.edu (M.C.); SHIMIZLS@mailbox.sc.edu (L.S.S.); 3 Department of Pathology, Uniformed Services University of the Health Sciences, Bethesda, MD 20814, USA; grant.kern.ctr@usuhs.edu

**Keywords:** small molecule, NSC243928, LY6K, cervical cancer, surface plasmon resonance

## Abstract

Elevated gene expression of Lymphocyte antigen 6K (LY6K) in cancer cells is associated with poor survival outcomes in multiple different cancer types including cervical, breast, ovarian, lung, and head and neck cancer. Since inhibition of LY6K expression inhibits cancer cell growth, we set out to explore whether pharmacological inhibition of LY6K could produce the same effect. We screened small molecule libraries for direct binding to recombinant LY6K protein in a surface plasmon resonance assay. We found that NSC243928 directly binds to the full-length and mature forms of LY6K and inhibits growth of HeLa cells that express LY6K. NSC243928 did not display binding with LY6D or LY6E. Our data demonstrate a first-time proof of principle study that pharmacological inhibition of LY6K using small molecules in cancer cells is a valid approach to developing targeted therapies against LY6K. This approach will be specifically relevant in hard-to-treat cancers where LY6K is highly expressed, such as cervical, pancreatic, ovarian, head and neck, lung, gastric, and triple-negative breast cancers.

## 1. Introduction

Human Lymphocyte Antigen 6 (LY6) gene family proteins are found clustered on chromosomes 6, 8, 11, and 19 [1]. The LY6 gene family is defined by the presence of a common LU domain present in the mature form of these small, glycosylated proteins, which are anchored to the extra-cellular side of the cell membrane by a glycosylphosphatidylinositol moiety. LY6 gene clusters contain 30 known genes and perform a variety of functions in spermatogenesis, neutrophil activation, complement-mediated lysis, and macrophage biology [1]. The LY6K gene is a member of this family, located on human chromosome 8q24. Human chromosome 8q24 is often amplified in many different types of cancers and has been studied in the context of Myc overexpression, a widely known resident of genetic loci 8q24 [2,3,4]. Recently, it has been shown that genes other than Myc on 8q24 play an important role in cancer progression [5]. High expression of LY6K mRNA is associated with poor survival outcomes in an array of solid cancers, including cervical, breast, head and neck, bladder, esophageal, lung, and colorectal cancer [6,7,8,9,10]. The expression of LY6K in normal cells is mainly found in testicular germ cells, where it plays a role in sperm cell migration [11]. LY6K knockout male mice show normal sexual behavior but are infertile, while LY6K knockout female mice are fertile [12]. The shRNA-mediated LY6K knockdown in cancer cells results in reduced tumor growth for in vivo tumor models [13]. Increased expression of LY6K is also associated with increased TGFβ signaling and tumor growth in vivo [13].

We envision that LY6K is an ideal target for drug development for women’s cancer since the molecule’s normal expression is limited to the testis. We sought to examine whether pharmacological inhibition of LY6K could be achieved using small molecules. We discovered one small molecule LY6K binder among the NCI’s small molecule library of approximately 2,600 small molecules. The identified small molecule, NSC243928, was previously known to have anti-cancer properties [14]. Here we report small molecule NSC243928 as a first-in-class LY6K binder which also acts as an inhibitor of cell growth. This study sets up the groundwork to discover novel drugs targeting LY6K to treat cancers with high LY6K expression.

## 2. Results

### 2.1. LY6K Is a Feasible Target to Develop Targeted Therapeutics

**Figure 1:**
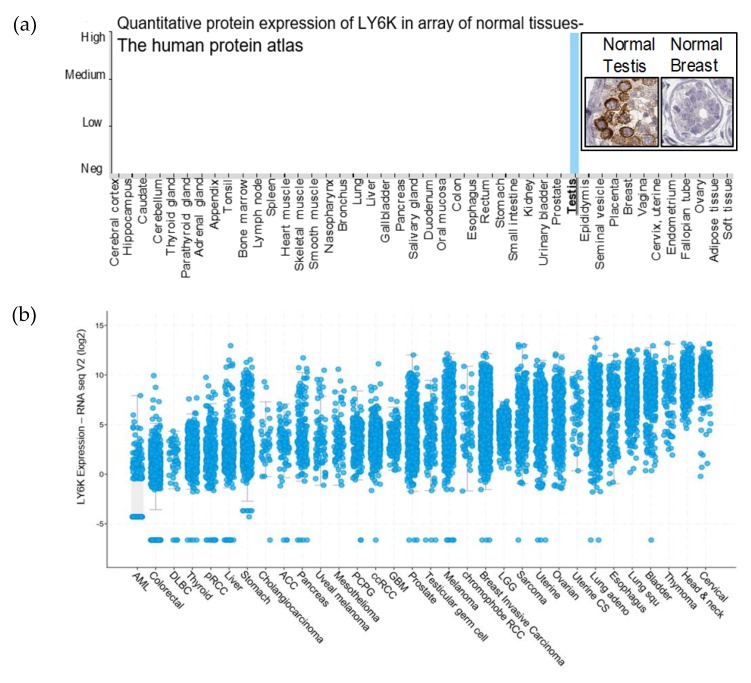
Lymphocyte antigen 6K (LY6K) is an ideal target for cancer therapeutics. (**a**) The Human Protein Atlas data shows quantification of Immunohistochemistry (IHC) using a nuclear magnetic resonance (NMR) validated, affinity purified using the PrEST-antigen as affinity ligand HPA017770 (Sigma) antibody for LY6K, in a panel of human normal tissues. The intensity of IHC labeling is shown on the Y-axis, and the X-axis shows the names of organs tested. In the inset, IHC images are shown from normal testis and breast using LY6K antibody. (**b**) cBioPortal data visualization using TCGA 2018 release data suggest high expression of LY6K in multiple cancer types.

Within a panel of normal tissues from the Human Protein Atlas, protein expression of LY6K was found to be limited to testis (Figure 1a) [15]. It was previously reported that LY6K RNA is not expressed within a panel of normal purified immune cells [16]. cBioPortal was used to visualize LY6K mRNA expression in The Cancer Genome Atlas (TCGA) pan-cancer datasets [17]. The 32 studies represented 32 cancer types containing RNA sequence data from 10,967 cancer samples in 10,953 patients. Every data point in each cancer type represents a unique sample. LY6K expression was elevated in many cancer types. The highest LY6K expression was seen in cervical, head and neck, bladder, esophageal, and breast cancer (Figure 1b).

### 2.2. Identification of a Small Molecule Binder of LY6K

**Figure 2:**
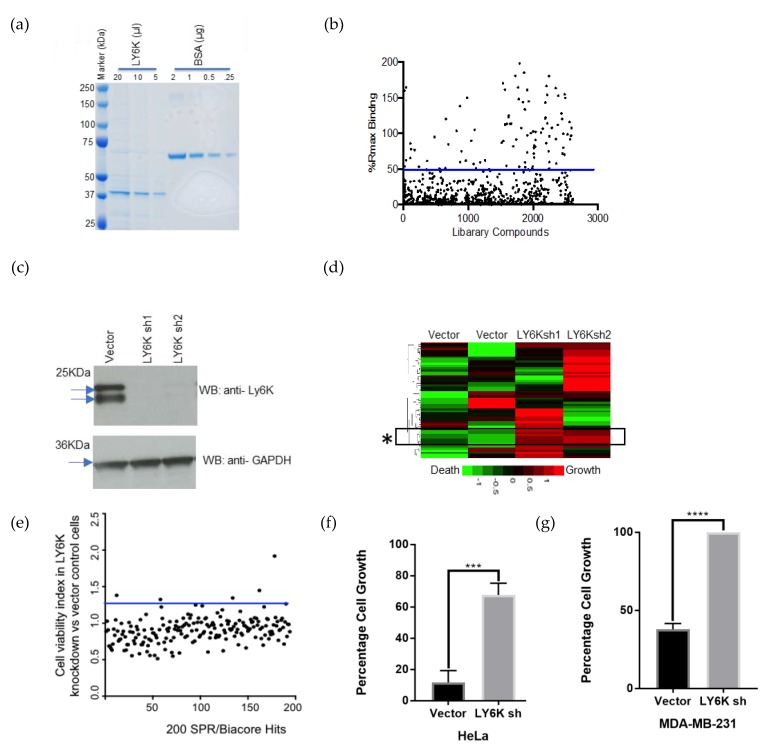
Identification of NSC243928 as a binder of LY6K. (**a**) Full-length GST-tagged LY6K protein was overexpressed in HEK 293 cells. GST sepharose beads were used to pull down the protein and were run on SDS-PAGE gel alongside BSA. (**b**) Surface plasmon resonance (SPR) data showing binding of NCI Developmental Therapeutics Program (DTP) small molecules to recombinant full-length LY6K. The Y axis is normalized to R_max_, which is the theoretical maximum signal that can be achieved based amount of protein immobilized on the chip surface. Blue line indicates the 50% threshold. (**c**) Western blot data validating LY6K knockdown compared to vector control in HeLa cells. A sheep anti-human LY6K antigen affinity-purified polyclonal antibody (R&D Systems #AF6648) was used. Two specific bands centered around 25 kDa are standard for this antibody for full-length LY6K [13]. (**d**) Differential drug assay on HeLa control and LY6K knockdown cells. A total of 180 compounds showed binding to LY6K over control. These compounds were tested in cell growth assay in duplicate. The subset of drugs with a lower activity corresponding to the loss of LY6K are indicated by ‘*’. (**e**) Cell viability fold change between LY6K shRNA and control HeLa cells. (**f**) HeLa Vector and sh2 Cell-Titer-Blue assay. HeLa cells were treated with 2 μM NSC243928. After 24 h, cell viability was assessed (*** indicates *p* < 0.001). (**g**) MDA-MB-231 Vector and sh2 Cell-Titer-Blue assay. MDA-MB-231 cells were treated with 2 μM NSC243928. Cell viability was assessed after 24 h of treatment (**** indicates *p* < 0.0001).

In order to identify small molecules that can directly bind to LY6K protein, we screened the NCI’s small molecule library in a surface plasmon resonance (SPR) experiment. Full-length LY6K was cloned in an N-terminal GST-tagged expression vector and transiently expressed in HEK-293T cells. Protein purification was done using Glutathione Sepharose 4B GST-tagged protein purification resin (Figure 2a). Purified recombinant full-length LY6K was immobilized on a CM5 Biacore chip and 2643 small molecules were injected one at a time at a single concentration over the protein-coated surface. We selected 180 small molecules with a binding signal above 50% of the expected theoretical binding value as primary hits (Figure 2b). Next, a Cell-Titer-Blue (CTB) functional cell viability assay was performed using the 180 small molecules on LY6K knockdown and control HeLa cells. Stable LY6K shRNA cells were generated by transduction of two different lentiviral shRNA constructs followed by a selection in puromycin. Western blotting confirmed the loss of endogenous LY6K protein in LY6K knockdown cells compared to vector control cells (Figure 2c, Figures S13 and S14; Table S2). Vector and LY6K knockdown cell lines were treated with the 180 NCI library drugs and the cell viability was determined using a CTB assay. A heat map depicting the percentage of dead cells in green and live cells in red was generated. The rectangle marked with an asterisk showed a subset of drugs that lost their cell death activity in a reproducible manner as seen in both LY6K knockdown cells (sh1 and sh2) (Figure 2d, asterisk). This data was replotted as a ratio between LY6K shRNA/control cells to identify the drug which had the biggest impact on its cell death activity due to loss of LY6K. For this experiment, cell viability was averaged. The average fold change in cell viability (cell viability index) was plotted in LY6K knockdown vs. control cells. Small molecule NSC243928, an anti-cancer agent [14], showed the highest loss in cell death activity in the LY6K knockdown cells (Figure 2e). Treatment with NSC243928 in LY6K knockdown sh2 cells led to an 8.5-fold decrease in cell death activity compared to vector control HeLa cells (Figure 2f).

To confirm the effect of NSC243928 in LY6K-expressing cells, we observed the parental control and sh2 LY6K knockdown in MDA-MB-231 cells, a triple negative breast cancer cell line. These cells were generated as previously described [13]. To test the effect of NSC243928 on cell growth, the cells were seeded into 96-well plates and treated with 2 μM drug for 24 h. A CTB assay was then performed according to manufacturer’s instructions to determine the cell growth. We observed that NSC243928 induces cell death in MDA-MB-231 control cells, while LY6K knockdown cells showed increased cell viability in the presence of NSC243928 (Figure 2g). These experiments indicate that NSC243928 may induce cell death through LY6K pathways.

### 2.3. Re-Synthesized NSC243928 Has Comparable Activity to the Library-Obtained Compound

**Figure 3:**
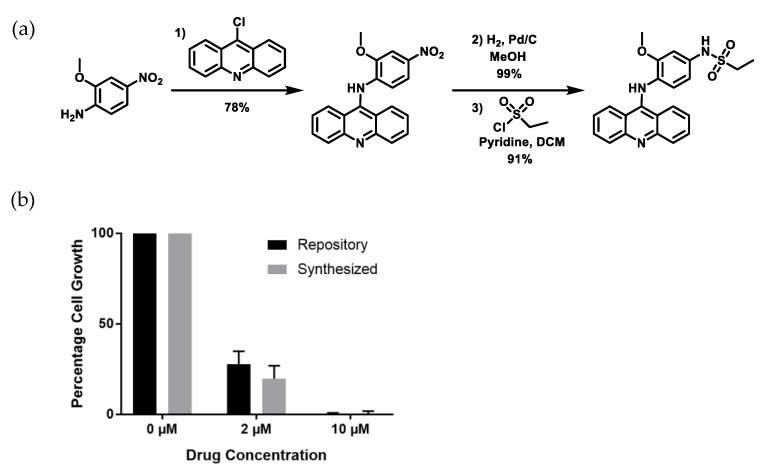
Synthesis and confirmation of NSC243928 activity. (**a**) Synthesis of NSC243928. Conditions: (1) 9-Chloroacridine, N-methyl-2-pyrrolidone (NMP), HCl, room temperature for 2 h. (2) H_2_, Pd/C, MeOH, room temperature for 2 h. (3) Ethane sulfonyl chloride, Pyridine, DCM, 0 °C to room temperature, overnight. (**b**) Cell death activity of repository (NCI) and synthesized (USC) compound in HeLa cells.

To further confirm the activity of NSC243928, the small molecule was resynthesized in three steps starting with an acid-catalyzed nucleophilic aromatic substitution of 9-chloroacridine with 2-methoxy-4-nitroaniline (Figure 3a). Reduction of the nitro group and subsequent treatment with ethane sulfonyl chloride yielded the desired product. Yellow NSC243928 crystals were used for structural and spectral studies to verify molecular purity and identity (Figure S1). The identity of the product was verified by ^1^H nuclear magnetic resonance spectroscopy, ^13^C nuclear magnetic resonance spectroscopy, single crystal X-ray diffraction, and liquid chromatography mass spectrometry (Figures S1–S9). Purity was monitored by liquid chromatography with two different detectors: UV (Figure S10) and charged aerosol detection (CAD, Figure S11). Resynthesized NSC243928 showed a similar IC50 to the repository compound in cell death assays in HeLa cells (Figure 3b).

### 2.4. NSC243928 Shows Specific Binding with the Mature Form of LY6K

**Figure 4:**
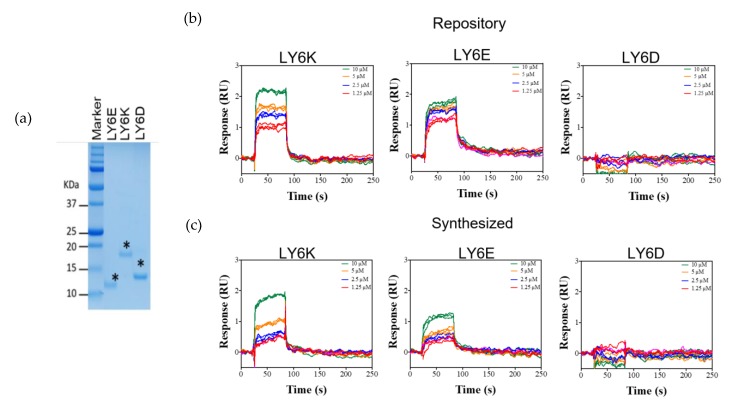
NSC243928 specifically binds to the mature form of LY6K. (**a**) The mature forms of LY6E, LY6D, and LY6K were cloned in pET24a N-term His-tag vector (Epoch Biosciences); expressed in BL21DE *Escherichia coli* (*E. coli*); purified using Ni-NTA resin; run on the 15% SDS-PAGE gel. The ‘*’ shows the specific LY6 protein bands of expected molecular weights. (**b**,**c**) Representative SPR sensorgrams for NSC243928 binding to LY6K, LY6E, and LY6D. NSC243928 shows a dose-dependent binding response to LY6K, whereas non-specific/no binding was observed for LY6E/LY6D, respectively; (**b**) NSC243928 obtained from NCI repository, (**c**) NSC243928 synthesized at USC.

LY6K and its family members LY6E and LY6D are glycosylphosphatidylinositol (GPI)-anchored proteins. It is predicted that the N-terminal and C-terminal sequences are cleaved off in the processed, mature protein. The mature form of the protein is processed for localization outside of the cell membrane via a GPI linker sequence. We tested whether NSC243928 binds to the mature form of LY6K and related genes LY6E and LY6D. For this experiment, the cDNA sequence encoding the mature forms of LY6K, LY6E, and LY6D were cloned into a pET-24a His-tag vector. Bacterially expressed proteins were purified using a Nickel-Nitrilotriacetic Acid (Ni-NTA) resin (Figure 4a). The NCI repository and USC synthesized versions of NSC243928 both showed concentration-dependent binding to the immobilized LY6K, but did not show concentration-dependent specific binding to the mature form of LY6E or LY6D (Figure 4b,c).

## 3. Discussion

**Figure 5:**
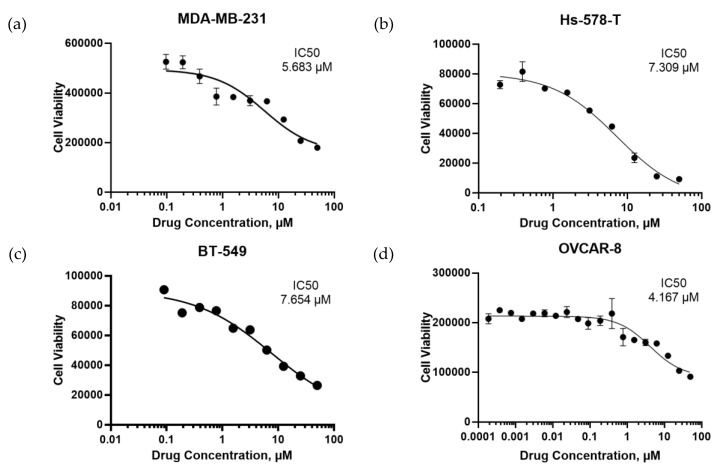
Cell death activity of NSC243298 in cancer cell lines. (**a**) MDA-MB-231 cells. (**b**) Hs-578-T cells. (**c**) BT-549 cells. (**d**) OVCAR-8 cells.

The bioactivity of NSC243928 was previously tested using a tumor cell line growth inhibition assay in a panel of cancer cell lines [14]. The compound has been found to be active in an in vitro cell death assay (Developmental Therapeutics Data, public Data). Our findings have confirmed that NSC243928 induces cell death in various cancer cell lines (Figure 5). Small molecule NSC243928 has previously been tested in an in vivo anti-tumor screen by the NCI on an L1210 leukemia model in BDF1 Syngeneic mice [14]. The average survival in this tumor model is 2–3 weeks. Treatment with NSC243928 increased survival benefit from 30% to 100% in a dose-dependent manner and was not found to be toxic up to 10 mg/kg/i.p. treatment [18]. 

Interestingly, NSC243928 was identified as one of 136 small molecules to inhibit the growth of ovarian cancer stem-like cells by high-throughput screening among 798 tested library compounds from the NCI repository [14]. It remains to be seen if LY6K is more active in stem-like cancer cells. LY6K is located on human chromosome 8, syntenic to mouse chromosome 15 which houses stem cell antigen-1 (Sca-1). Sca-1 is a cancer stem cell marker and plays a causal role in disrupted TGFβ signaling in mammary mouse tumor models [19]. While there is no human ortholog for Sca-1, several other genes on human chromosome 8 exist, including LY6E and LY6K [20]. So far, the stem cell-like properties of these genes have not been demonstrated. The mechanism of action for NSC243928 has not yet been fully elucidated. Currently, we are conducting *in vivo* experiments to understand the LY6K-NSC243928 molecular mechanism of action leading to cancer cell death. More work is needed to understand how LY6K can be used as a potential therapeutic target for precision medicine cancer treatment, using this new biomarker to design more effective treatment plans. In the future, pharmacological inhibition of LY6K may be a viable option for novel cancer treatment. As the normal function of LY6K is limited to normal testis, it is reasonable to anticipate limited toxicity associated with LY6K-targeted cancer treatment.

## 4. Materials and Methods

### 4.1. Plasmids

N-terminal GST-tagged LY6K was cloned into a mammalian expression vector pEBG with the EF1 promoter. The parent vector was a gift from Dr. Mayer, UCHS, CT and described previously [21]. N-terminal His-tagged mature forms of human LY6K, LY6D, and LY6E were gene synthesized in pET-24a vector (Epoch Biosciences, Bothell, WA, USA).

### 4.2. Mammalian Cell Culture and Transfection

Expression of mammalian LY6K protein in HEK-293T: HEK-293T cells were obtained from ATCC. Cells were cultured in a 37 °C incubator with 5% CO_2_. Cells were seeded in 100 mm cell culture dishes in DMEM with 10% FBS. Cells were transfected with the pEBG-GST-LY6K plasmid using PolyJet (SignaGen, Rockville, MD, USA) according to manufacturer’s protocol. The transfected cells were maintained in culture for 27 h for protein production. After 27 h, the cells were collected, and protein lysates were prepared using RIPA lysis buffer containing 1% glycerol.

LY6K shRNAs and cell lines: LY6K sh1 (Cat# TRCN0000117952), LY6K sh2 (Cat# TRCN0000117953), shRNAs cloned into pLKO.1 were obtained from Sigma Inc. Lentivirus was produced in 293T cells by cotransfection of the pMD2.g and VSVG vectors. At 24 h after transfection, the medium was replaced, and virus was collected. Cells were infected with lentivirus for 24 h in the presence of 4 mg/mL of polybrene, and selection carried out with 1 µg/mL of puromycin. The Hela cells were obtained by Georgetown University core facility. The cell line was authenticated by short random repeat DNA sequencing. Cells were propagated and stored in multiple vials as recommended. Each vial was cells were used and discarded within six months.

### 4.3. Purification of Full-Length GST-LY6K Protein from Mammalian Cells

The cleared protein lysate was subjected to pull down using GST beads (GE Healthcare, Chicago, IL, USA) per manufacturer’s instructions. The supernatant was incubated with a 50% slurry of activated Glutathione beads overnight at 4 °C. The beads were then centrifuged at 600 g and washed four times with PBS containing 1% Triton X-100, then once with PBS. The proteins were then eluted with an elution buffer containing 40 mM reduced Glutathione (Sigma, St. Louis, MO, USA) solution, 1% Triton X-100, and 1 mM dithiothreitol (DTT). The eluates were analyzed by SDS-PAGE and stained with Coomassie Brilliant Blue. Glutathione was removed from the eluted proteins via buffer exchange with HBS-P buffer (0.01 M HEPES pH 7.4, 0.15 M NaCl, 3 mM EDTA, 0.005% *v/v* Surfactant P20) using a 3K Amicon Ultra Centrifugal Filter Unit (Millipore, Burlington, MA, USA) for compatibility with Biacore. The eluates were then split into 25 µL aliquots and stored at −80 °C until use.

### 4.4. Purification of Mature Form of LY6K, LY6D, and LY6E Proteins from E. coli

Plasmids containing the sequences for N-terminal His-tagged mature LY6 proteins were transformed into BL21 (DE3) *E. coli* via heat shock. The cells were grown, and protein expression was induced with Isopropyl β-D-1-thiogalactopyranoside (IPTG). Following centrifugation, the cell pellet was collected and treated with urea buffer to obtain a protein lysate. A Ni-NTA resin (Roche, Basel, Switzerland) was used in a batch purification method and protein was eluted using imidazole according to the manufacturer’s instructions. Protein purity was assessed by SDS-PAGE on a 15% acrylamide gel.

### 4.5. Small Molecule Library and Preparation of Master Drug Plates

Oncology set, Diversity Set IV, Mechanistic set II, and Natural Products Set II small molecule libraries containing a total of 2,643 small molecules were obtained from the NCI Developmental Therapeutics Program (DTP). These molecules were then diluted to 1 mM in DMSO. All small molecules, except those from Natural Products Set II, were further diluted to a final concentration of 50 µM in PBS (20 mM phosphate buffer pH 7.4, 137 mM KCl, 2.7 mM NaCl) containing 0.05% *v/v* surfactant P-20 (termed as PBS-P hereafter) supplemented at 5% *v/v* DMSO and allocated in eight 384 microplates. Small molecules from Natural Products Set II were further diluted to a final concentration of 50 µM in PBS-P supplemented with 4.75% *v/v* DMSO and 0.25% *v/v* glycerol and allocated in a separate microplate.

### 4.6. Surface Plasmon Resonance

Biacore T200 was used for all in vitro SPR screening experiments. To test the ability of the small molecules to bind to LY6K-GST, LY6K-GST was covalently immobilized to flow cell 2 (FC2) of a CM5sensor chip to ≈10,000 RU (response units) using standard amine coupling chemistry in the presence of 10 mM sodium acetate buffer at pH 4.0. A randomly chosen negative control protein (CD99) was also captured on FC4 of the same sensor chip using standard biotin-neutravidin coupling chemistry. HBS-P (10 mM Hepes pH 7.4, 150 mM NaCl, 0.05% *v/v* surfactant P20) was used as the immobilization/capture running buffer.

FC1 was used as the reference for FC2 and FC3 was used as the reference for FC4. The reference FCs had the same coupling chemistry as the active FCs (FC2 and FC4) but did not have proteins. Small molecules from plates one to nine were injected over these proteins for a period of 60 s at a flow rate of 30 µL/min. After each injection, compounds were allowed to dissociate for 120 s before starting the next compound injection cycle. After every 40 compound injections, multiple blank buffer (PBS-P +5% DMSO or PBS-P +4.75% DMSO +0.25% glycerol) injections were made. Fresh sensor chips and proteins were used for each 384-well plate.

Compound-LY6K and compound-control-protein binding levels were determined after subtracting the non-specific binding to the reference surfaces and response values for the blank buffer injections (double reference subtraction). Theoretical maximum binding levels (R_max_) were calculated, and each molecule’s real binding percentage was compared to its corresponding R_max_ value. Compounds binding to the negative control protein more than the target protein were eliminated from further evaluation. Compounds binding to LY6K with 50% or more of the R_max_ were selected as primary hits. Compounds that showed more than 200% R_max_ were considered non-specific aggregates and eliminated from further evaluation. Dose response experiments for LY6D, LY6E, and LY6K were immobilized on FC2, FC3, and FC4 using a standard amine coupling chemistry in the presence of 10 mM sodium acetate buffer at pH values 4.5, 5.5, and 4.0 to levels of ≈800–950 RU, ≈900–1,700 RU, and ≈1,400–3,400 RU, respectively.

### 4.7. Synthesis of NSC243928

Synthesis of NSC243928 was performed in three steps. 

First step—synthesis of N-(2-methoxy-4-nitrophenyl)acridin-9-amine (1): 2-methoxy-4-nitroaniline (0.842 g, 5.01 mmol) and 9-chloroacridine (0.973 g, 4.55 mmol) were dissolved in N-methyl-2-pyrrolidone (NMP) (17 mL) and two drops of concentrated HCl were added. The reaction was stirred at r.t. for 4 h. Afterwards, ethyl acetate (EtOAc) (≈50 mL) was added to form a precipitate, which was collected by filtration. The solid was redissolved in methanol (MeOH) at 45 °C, reprecipitated with EtOAc, and filtered to yield a yellow solid (1.22 g, 78%) [22,23]. ^1^H NMR (300 MHz, MeOD) δ 8.23 (d, J = 8.8 Hz, 2H), 8.07–8.00 (m, 6H), 7.67 (d, J = 8.6 Hz, 1H), 7.57–7.52 (m, 2H), 3.69 (s, 3H). ^13^C NMR (100 MHz, MeOD) δ 157.46, 154.51, 148.59, 141.49, 137.25, 136.80, 126.69, 126.17, 125.97, 120.45, 118.04, 116.48, 108.79, 56.96.

Second step—synthesis of N1-(acridin-9-yl)-2-methoxybenzene-1,4-diamine (2): 1 (0.800 g, 2.32 mmol) and a catalytic amount of Pd/C were added to 100 mL MeOH in a pressure flask. The resulting suspension was agitated under 50 psi of H_2_ for 2 h. The suspension was then filtered to remove the Pd/C. The filtrate was evaporated under reduced pressure to yield the crude product as a yellow solid (0.730 g, 99%), which was carried on without further purification [22,23]. ^1^H NMR (300 MHz, MeOD) δ 8.26 (d, J = 8.7 Hz, 2H), 7.94–7.91 (m, 2H), 7.84 (t, J = 7.9 Hz, 2H), 7.39 (t, J = 7.7 Hz, 2H), 7.16 (d, J = 8.3 Hz, 1H), 6.50–6.44 (m, 2H), 3.54 (s, 3H).

Third step—synthesis of NSC243928: N1-(acridin-9-yl)-2-methoxybenzene-1,4-diamine from step 2 (0.228 g, 0.723 mmol) and dry pyridine (0.086 mL, 1.06 mmol) were added to 3.6 mL of dry dichloromethane (DCM). Ethane sulfonyl chloride (0.206 mL, 2.17 mmol) was then added to the reaction mixture dropwise. Due to toxicity of ethane sulfonyl chloride, the addition was carried out in a fume hood set to an emergency setting with a face velocity around 500 feet per minute and the following PPE was worn: neoprene gloves on top of nitrile gloves, lab coat, face shield. Any materials that came in contact with the reagent were then set in an ice water bath to destroy any residual ethane sulfonyl chloride before being disposed of. The mixture was stirred at room temperature overnight. MeOH was added to destroy any excess ethane sulfonyl chloride and the solvent was evaporated under reduced pressure. The crude mixture was dry loaded on a silica gel column and the product was isolated using the following gradient (100% DCM to 90:10 DCM:MeOH) to yield a yellow solid (0.268 g, 91%) [24]. ^1^H NMR (300 MHz, MeOD) δ 8.01 (d, J = 8.5 Hz, 2H), 7.67–7.60 (m, 4H), 7.18–7.13 (m, 2H), 6.99 (s, 1H), 6.81 (s, 2H), 3.69 (s, 3H), 3.14 (q, J = 7.4 Hz, 2H), 1.35 (t, J = 7.4 Hz, 3H). ^13^C NMR (100 MHz, MeOD) δ 153.53, 152.14, 144.84, 135.59, 135.50, 132.76, 126.75, 123.19, 122.20, 122.03, 119.76, 114.60, 106.61, 56.16, 46.25, 8.48.

### 4.8. X-Ray Structure Determination, C_22_H_21_N_3_O_3_S·H_2_O (NSC24392**8**)

X-ray intensity data from an orange wedge-shaped crystal were collected at 100(2) K using a Bruker D8 QUEST diffractometer equipped with a PHOTON-100 CMOS area detector and an Incoatec microfocus source (Mo Kα radiation, λ = 0.71073 Å). The raw area detector data frames were reduced and corrected for absorption effects using the Bruker APEX3, SAINT+ and SADABS programs [25,26]. The structure was solved with SHELXT [27,28]. Subsequent difference Fourier calculations and full-matrix least-squares refinement against F2 were performed with SHELXL-2018 using OLEX2 [28,29].

### 4.9. Cell Death Assays

For cell death assay, cells were plated in a black walled 96-well plate and treated with the indicated concentration of drugs for 24 h. Cell-Titer-Blue reagent was added, and the Cytation 5 microplate reader (BioTek, Winooski, VT, USA) was used to measure fluorescence intensity (excitation 560(10) nm, emission 590(20) nm) according to manufacturer’s instructions.

## 5. Conclusions

In this report, we show that NSC243928 interacts specifically with LY6K in a dose-dependent manner. We used a full-length protein GST-LY6K fusion isolated from mammalian cells to identify small molecule LY6K binders, from which we selected NSC243928 for additional analysis. Furthermore, we found out that NSC243928 also shows binding with the mature form of LY6K fused with a His-tag. NSC243928 did not show specific binding with other LY6 family proteins, LY6E and LY6D. These data suggest that it is feasible to find specific small molecules that bind to LY6 proteins.

## 6. Patents

A use of patent application for NSC243928 and its derivatives for binding and targeting LY6K has been submitted.

## Supplementary Materials

The following are available online at https://www.mdpi.com/2072-6694/12/2/509/s1, Figure S1: Structural and spectral analysis of NSC243928. Figure S2: ^1^H NMR (300 MHz) of **1** in CD_3_OD. Figure S3: ^13^C NMR (100 MHz) of **1** in CD_3_OD. Figure S4: ^1^H NMR (300 MHz) of intermediate **2** in CD_3_OD. Figure S5: ^1^H NMR (300 MHz) of NSC243928 in CD_3_OD. Figure S6: ^13^C NMR (100 MHz) of NSC243928 in CD_3_OD. Figure S7: LC/MS chromatogram of NSC243928 showing the presence of four peaks. Figure S8: MS of peaks A ([M + H]^+^ = 210 g/mol) and B ([M + H]^+^ = 408 g/mol). Figure S9: MS of peaks C ([M + H]^+^ = 350 g/mol) and D ([M + H]^+^ = 442 g/mol). Figure S10: LC/UV chromatogram of NSC243928 (95.52% purity). Figure S11: LC/CAD chromatogram of NSC243928 (95.95% purity). Figure S12: LC/CAD chromatogram of control (blank). Figure S13: Whole Western blot images. Figure S14: LY6K/GAPDH intensity ratios. Table S1: Crystal data for NSC243928. Table S2: Western blot densitometry data.
