# Supplementary material for: Small Molecule Binds with Lymphocyte Antigen 6K to Induce Cancer Cell Death
Source: Cancers (Basel). 2020 Feb 22;12(2):509. doi: 10.3390/cancers12020509 (PMC7072568; doi:10.3390/cancers12020509)
Supplement: Supplementary file 1 [file cancers-12-00509-s001.pdf]

Supplementary Materials

Small Molecule Binds with Lymphocyte Antigen 6K to Induce Cancer Cell Death

Senyi Benti, Purushottam B. Tiwari, Dustin W. Goodlett, Leily Daneshian, Grant B. Kern, Mark D. Smith, Aykut Uren, Maksymilian Chruszcz, Linda S. Shimizu and Geeta Upadhyay

Supplementary Methods

Method for NMR: ^{1}H NMR and ^{13}C NMR spectra were recorded on Bruker Avance III-HD spectrometers (300–400 MHz). Chemical shifts are reported as (δ ppm) with the corresponding integration values, while coupling constants (J-values) are reported in hertz (Hz).

Method for HPLC: LC was carried out using a Thermo Scientific Dionex Ultimate 3400 RS HPLC system with a Waters XBridge C18 3.5 µm particles; 2.1 mm × 100 mm column. MS data was collected using a Waters QTof API US, quadrupole-time-of-flight mass spectrometer. DAD data was collected using an Agilent 1100 Model 1315B UV diode array detector. CAD data was collected using a Thermo Scientific Corona Veo RS charged aerosol detector.

List of Figures

Figure S1: Structural and spectral analysis of NSC243928.
Scheme S1: Synthesis of NSC243928.
Figure S2: ^{1}H NMR (300 MHz) of **1** in CD_3OD.
Figure S3: ^{13}C NMR (100 MHz) of **1** in CD_3OD.
Figure S4: ^{1}H NMR (300 MHz) of intermediate **2** in CD_3OD.
Figure S5: ^{1}H NMR (300 MHz) of NSC243928 in CD_3OD.
Figure S6: ^{13}C NMR (100 MHz) of NSC243928 in CD_3OD.
Figure S7: LC/MS chromatogram of NSC243928 showing the presence of four peaks.
Figure S8: MS of peaks A ($[M + H]^+$ = 210 g/mol) and B ($[M + H]^+$ = 408 g/mol).
Figure S9: MS of peaks C ($[M + H]^+$ = 350 g/mol) and D ($[M + H]^+$ = 442 g/mol).
Figure S10: LC/UV chromatogram of NSC243928 (95.52% purity).
Figure S11: LC/CAD chromatogram of NSC243928 (95.95% purity).
Table S1: Crystal data for NSC243928.
Figure S12: LC/CAD chromatogram of control (blank).
Figure S13: Whole western blot images.
Table S2: Western blot densitometry data.
Figure S14: LY6K/GAPDH intensity ratios.

Structural Studies of NSC243928

The crystalized NSC243928 was used for structural studies (Supplemental Figure 1A). The compound crystallizes in the triclinic system. The space group P-1 (No. 2) was confirmed by structure solution. The asymmetric unit consists of one $C_{22}H_{21}N_3O_3S$ molecule and one H_2O molecule. All non-hydrogen atoms were refined with anisotropic displacement parameters. Hydrogen atoms bonded to carbon were located in difference Fourier maps before being placed in geometrically idealized positions. These hydrogen atoms were included as isotropically refined riding atoms with d(C-H) = 0.95 Å for arene hydrogen atoms, d(C-H) = 0.99 Å for methylene hydrogen atoms and d(C-H) = 0.98 Å for methyl hydrogens. Methyl hydrogens were allowed to rotate as a rigid group to the orientation of maximum observed electron density. Water hydrogen atoms and those bonded to nitrogen were located and refined freely with isotropic displacement parameters, with O-H and N-H distances restrained to be similar to those of the same kind. The largest residual electron density peak in the final difference map is 0.43 e-/Å3, located 0.93 Å from S1 (Figure S1B).

In solution (10% DMSO/H_2O), NSC243928 displays an absorption band at λ_{max} = 330 nm. Excitation at this wavelength leads to three emission peaks in the visible region (417 nm, 440 nm, and 466 nm) (Figure S1C).

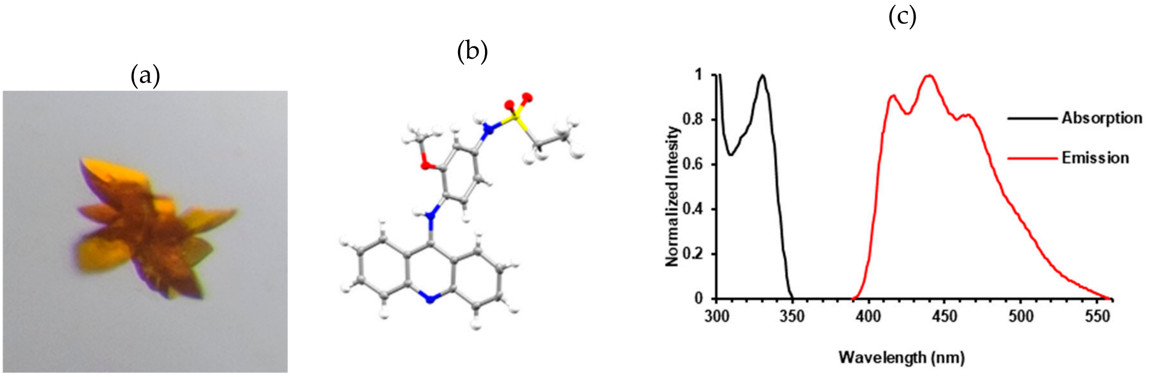

Figure S1. Structural and spectral analysis of NSC243928. (**a**) Orange crystals of NSC243928 monohydrate. (**b**) Molecular structure. (**c**) Absorption and emission spectra (λ_{exc} = 330 nm) of NSC243928 (58 µM in 10% DMSO aqueous solution).

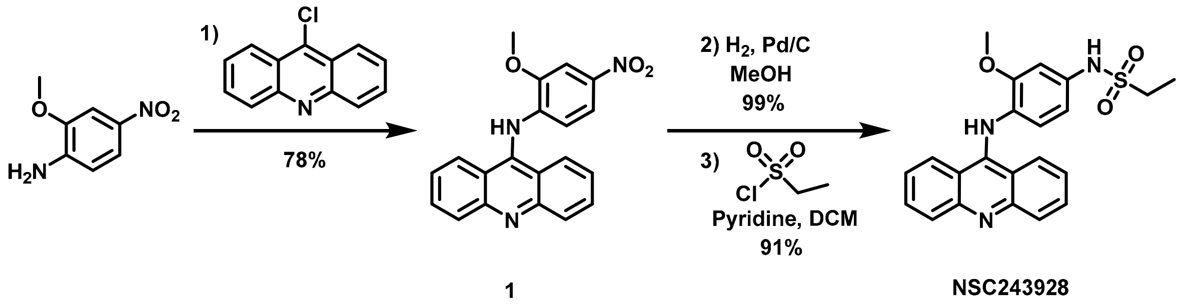

Scheme S1: Synthesis of NSC243928. Reagents and conditions: 9-chloroacridine was substituted with 2-methoxy-4-nitroaniline in NMP using a catalytic amount of concentrated HCl to produce **1**. The nitro group of **1** was then reduced in the presence of H_2 using a catalytic amount of Pd/C in MeOH. The resulting amine was treated with ethane sulfonyl chloride in the presence of dry pyridine in dry DCM to yield the resulting NSC243928 product.

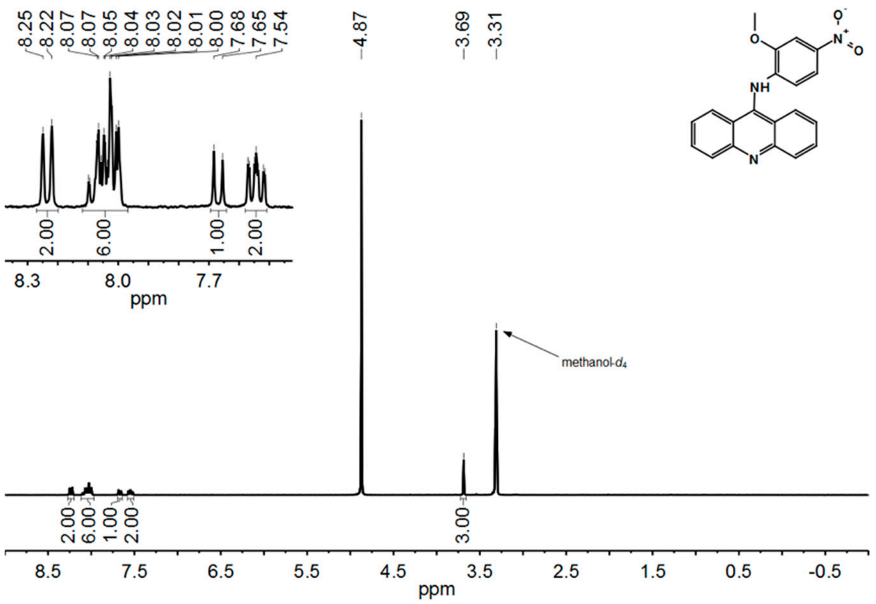

Figure S2. ^{1}H NMR (300 MHz, CD_3OD) of **1**.

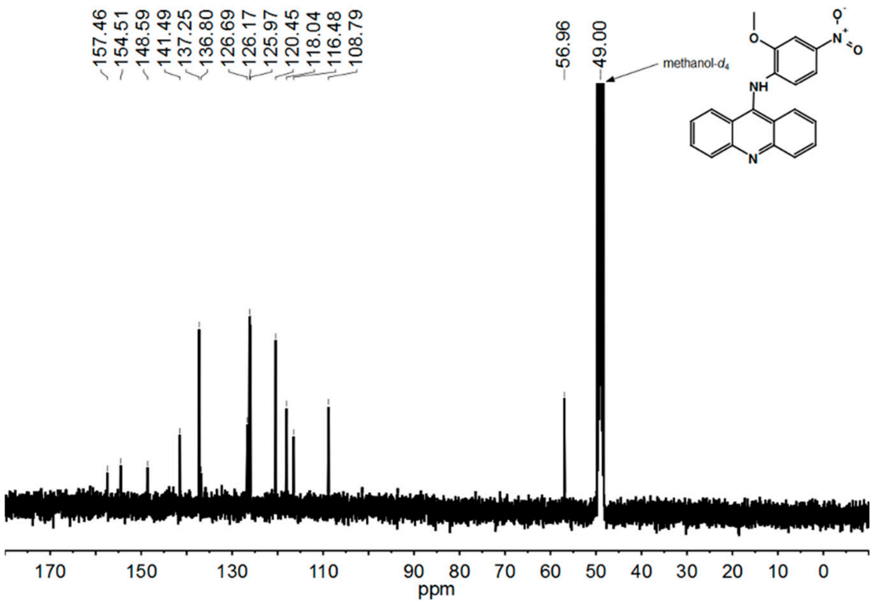

Figure S3. ^{13}C NMR (100 MHz, CD_3OD) of **1**.

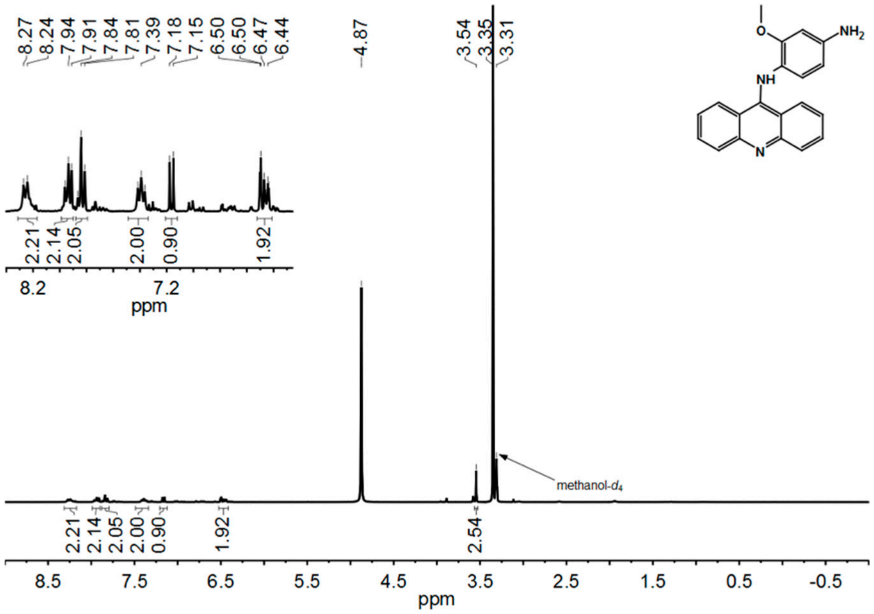

Figure S4. ^{1}H NMR (300 MHz, CD_3OD) of intermediate **2**.

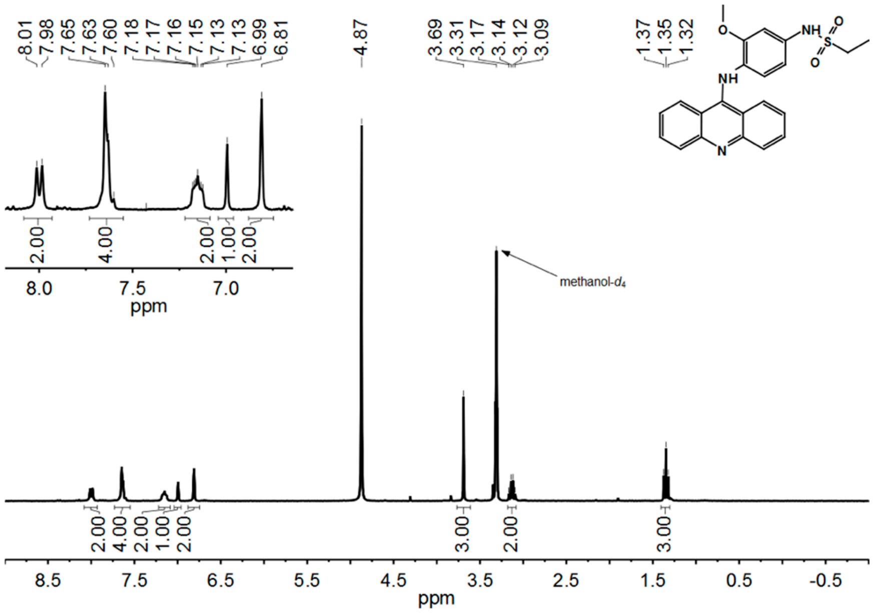

Figure S5. ^1H NMR (300 MHz, CD_3OD) of NSC243928.

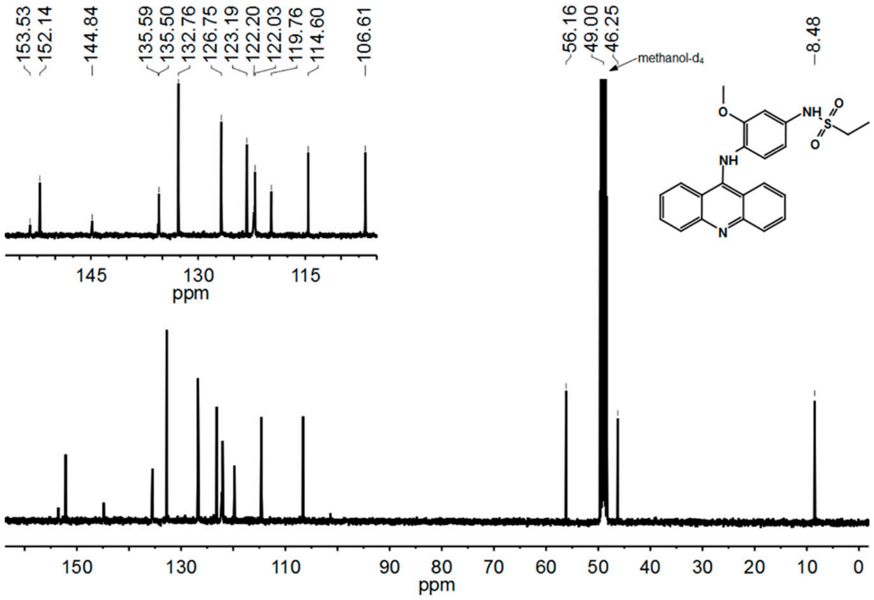

Figure S6. ^{13}C NMR (100 MHz, CD_3OD) of NSC243928.

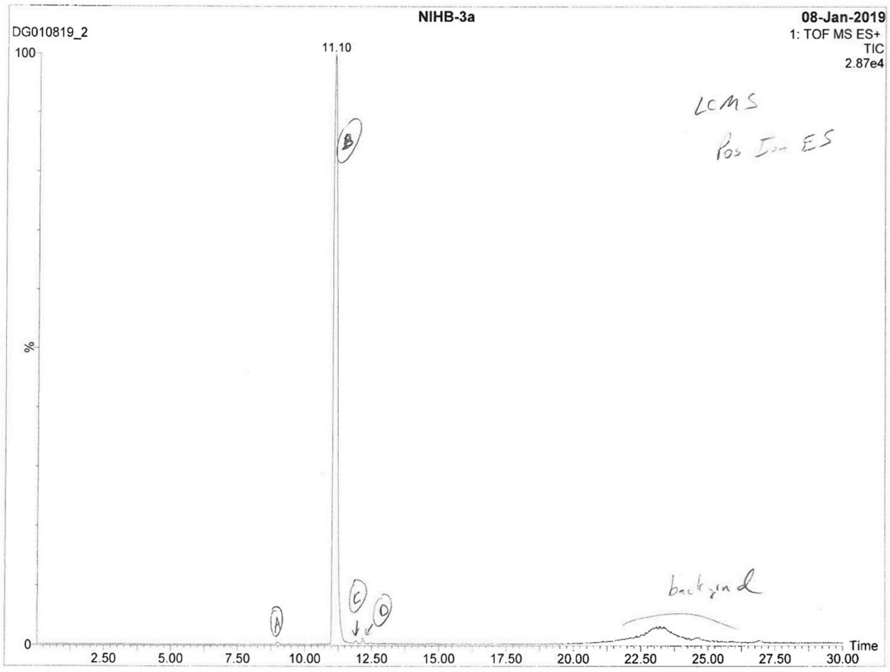

Figure S7. LC/MS chromatogram of NSC243928 revealing the presence of 4 peaks.

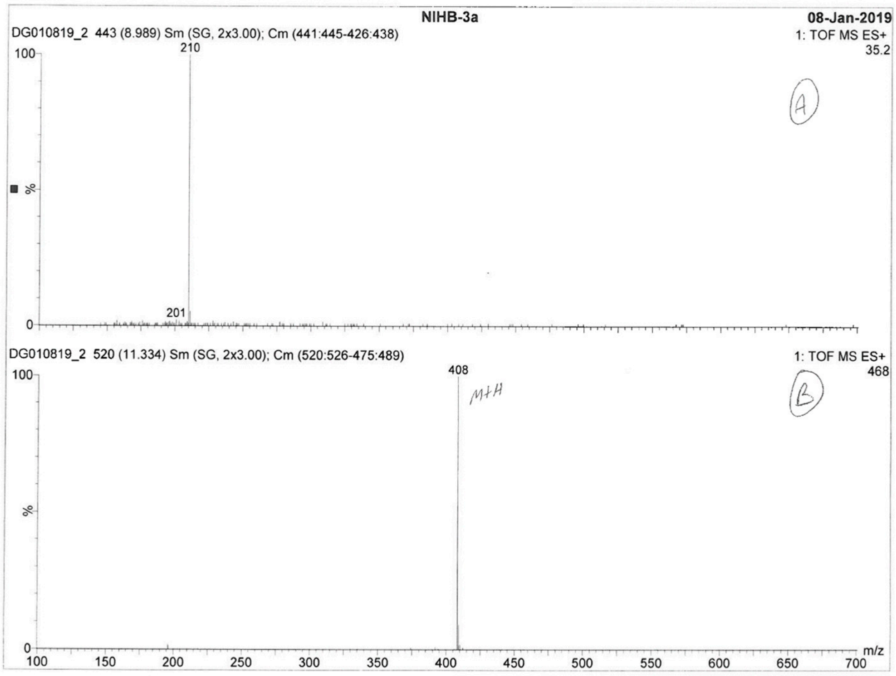

Figure S8. MS of peaks A and B from the LC/MS chromatogram. B has the correct mass for NSC243928 $[M + H]^+$).

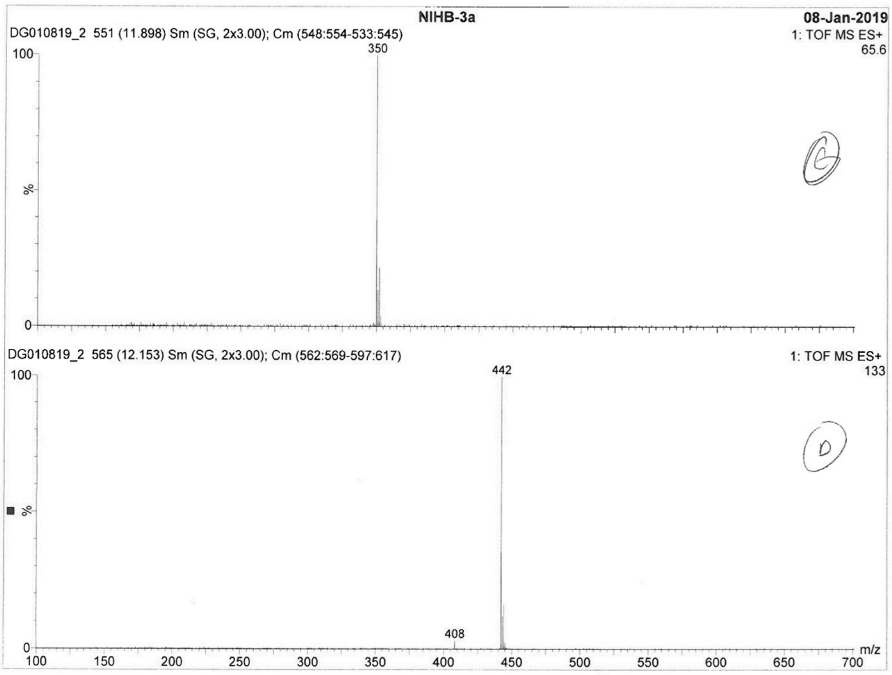

Figure S9. MS of peaks C and D from the LC/MS chromatogram.

Instrument:Ultimate3000 Sequence:routine LCMS JAN 2019 Page 1 of 1

Chromatogram and Results

Injection Details

Injection Name:	**NIHB-3a**	*Run Time (min):*	**36.99**
Vial Number:	**BE8**	*Injection Volume:*	**3.00**
Injection Type:	**Unknown**	*Channel:*	**DAD_Signal_A**
Calibration Level:		*Wavelength:*	**n.a.**
Instrument Method:	**patheongrad**	*Bandwidth:*	**n.a.**
Processing Method:	**New Processing Method**	*Dilution Factor:*	**1.0000**
Injection Date/Time:	**08/Jan/19 14:13**	*Sample Weight:*	**1.0000**

Chromatogram

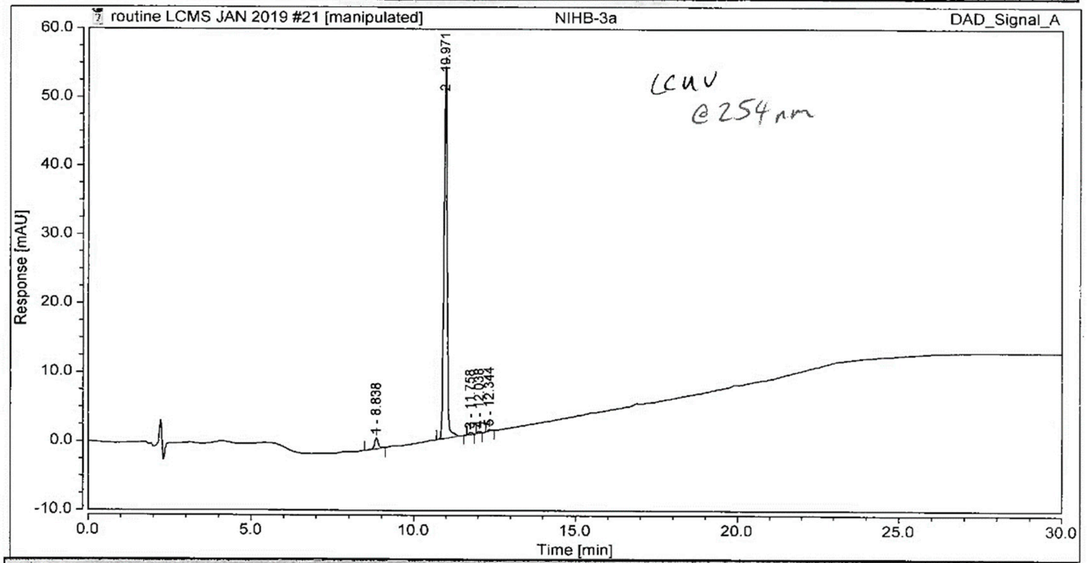

Integration Results

No.	Peak Name	Retention Time min	Area mAU*min	Height mAU	Relative Area %	Relative Height %	Amount n.a.
1		8.838	0.193	1.594	2.99	2.82	n.a.
2		10.971	6.171	54.013	95.52	95.52	n.a.
3		11.758	0.039	0.370	0.60	0.65	n.a.
4		12.038	0.032	0.308	0.50	0.54	n.a.
5		12.344	0.025	0.262	0.38	0.46	n.a.
**Total:**			**6.460**	**56.547**	**100.00**	**100.00**	

Default DAD/Integration

Chromeleon (c) Dionex Version 7.2.2.6394

Figure S10. LC/UV chromatogram at 254 nm of NSC243928 suggesting a purity of 95.5%.

Instrument:Ultimate3000 Sequence:routine LCMS JAN 2019 Page 1 of 1

Chromatogram and Results

Injection Details

Injection Name:	**NIHB-3a**	*Run Time (min):*	**42.00**
Vial Number:	**BE5**	*Injection Volume:*	**5.00**
Injection Type:	**Unknown**	*Channel:*	**CAD_1**
Calibration Level:		*Wavelength:*	**n.a.**
Instrument Method:	**patheongradcad**	*Bandwidth:*	**n.a.**
Processing Method:	**New Processing Method**	*Dilution Factor:*	**1.0000**
Injection Date/Time:	**11/Jan/19 12:08**	*Sample Weight:*	**1.0000**

Chromatogram

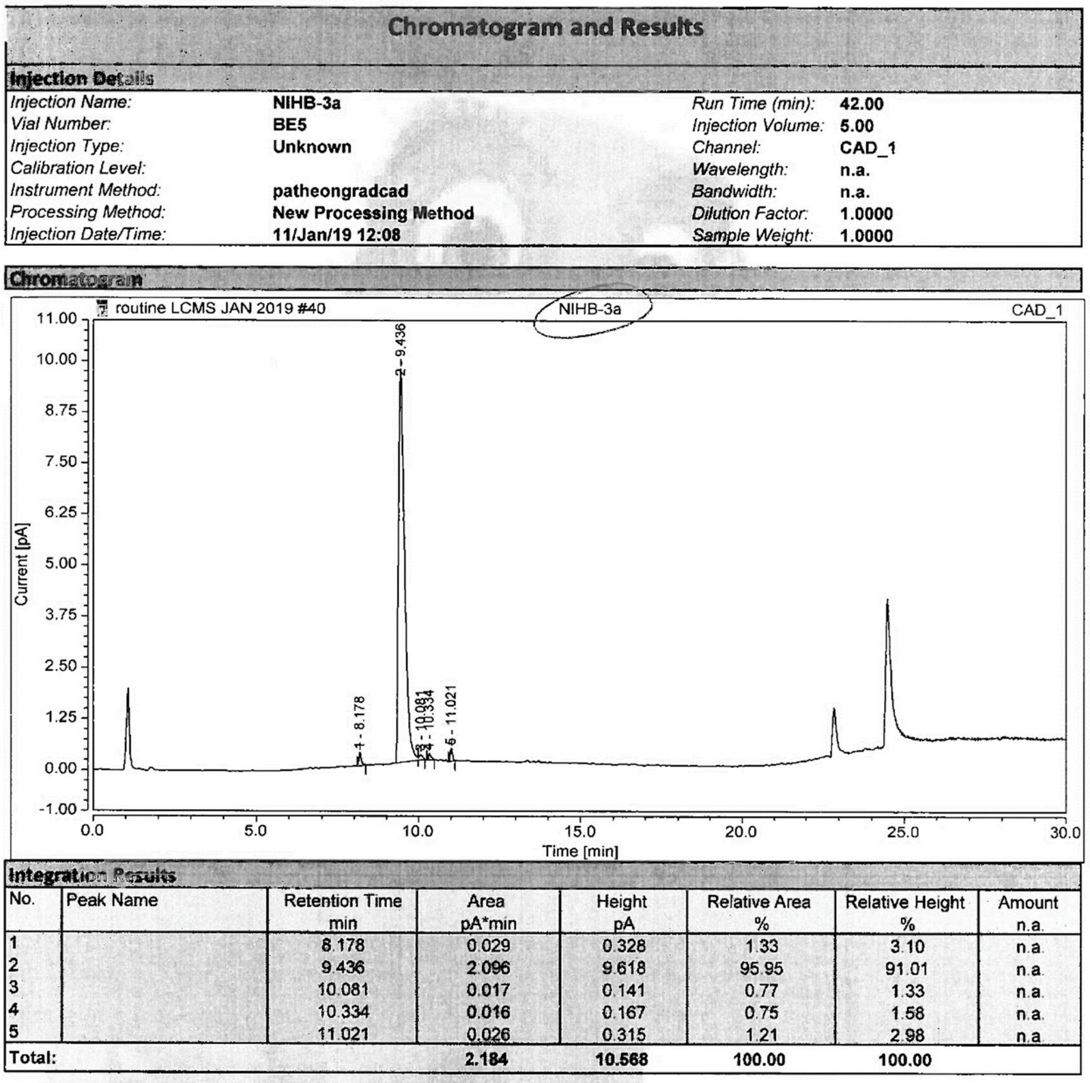

Integration Results

No.	Peak Name	Retention Time min	Area pA*min	Height pA	Relative Area %	Relative Height %	Amount n.a.
1		8.178	0.029	0.328	1.33	3.10	n.a.
2		9.436	2.096	9.618	95.95	91.01	n.a.
3		10.081	0.017	0.141	0.77	1.33	n.a.
4		10.334	0.016	0.167	0.75	1.58	n.a.
5		11.021	0.026	0.315	1.21	2.98	n.a.
**Total:**			**2.184**	**10.568**	**100.00**	**100.00**	

Default DAD/Integration

Chromeleon (c) Dionex
Version 7.2.2.6394

Figure S11. LC/CAD chromatogram of NSC243928 suggesting a purity of 95.9%.

Instrument:Ultimate3000 Sequence:routine LCMS JAN 2019 Page 1 of 1

Chromatogram and Results

Injection Details

Injection Name:	**BLANK**	*Run Time (min):*	**42.00**
Vial Number:	**BD1**	*Injection Volume:*	**5.00**
Injection Type:	**Unknown**	*Channel:*	**CAD_1**
Calibration Level:		*Wavelength:*	**n.a.**
Instrument Method:	**patheongradcad**	*Bandwidth:*	**n.a.**
Processing Method:	**New Processing Method**	*Dilution Factor:*	**1.0000**
Injection Date/Time:	**11/Jan/19 11:26**	*Sample Weight:*	**1.0000**

Chromatogram

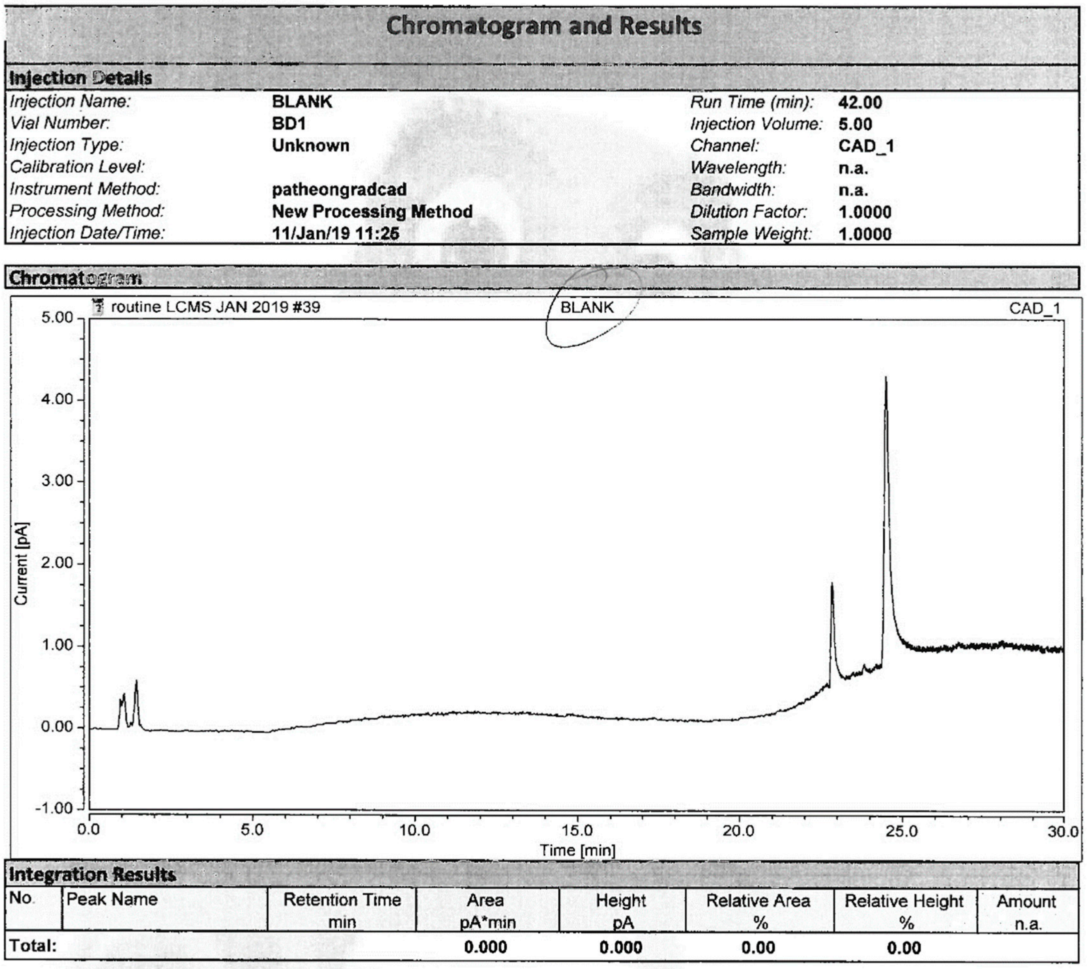

Integration Results

No.	Peak Name	Retention Time min	Area pA*min	Height pA	Relative Area %	Relative Height %	Amount n.a.
**Total:**			**0.000**	**0.000**	**0.00**	**0.00**	

Default DAD/Integration

Chromeleon (c) Dionex
Version 7.2.2.6394

Figure S12. LC/CAD chromatogram of the Blank.

Table S1. Crystal data for NSC243928.

Empirical formula	$C_{22}H_{23}N_3O_4S$
Formula weight	425.49
Temperature/K	100(2)
Crystal system	triclinic
Space group	P-1
a/Å	9.5341(6)
b/Å	11.0364(9)
c/Å	11.0438(9)
α/°	66.285(4)
β/°	82.429(4)
γ/°	66.795(4)
Volume/Å^3	977.36(13)
Z	2
ρ_{calc}g/cm^3	1.446
μ/mm^{-1}	0.202
F(000)	448.0
Crystal size/mm^3	0.1 × 0.08 × 0.07
Radiation	MoKα (λ = 0.71073)
2Θ range for data collection/°	4.35 to 52.836
Index ranges	-11 ≤ h ≤ 11, -13 ≤ k ≤ 13, -13 ≤ l ≤ 13
Reflections collected	11172
Independent reflections	3973 [R_{int} = 0.0495, R_{sigma} = 0.0580]
Data/restraints/parameters	3973/2/309
Goodness-of-fit on F^2	1.010
Final R indexes [I>=2σ (I)]	R_1 = 0.0486, wR_2 = 0.1104
Final R indexes [all data]	R_1 = 0.0791, wR_2 = 0.1246
Largest diff. peak/hole/e Å^{-3}	0.43/-0.52

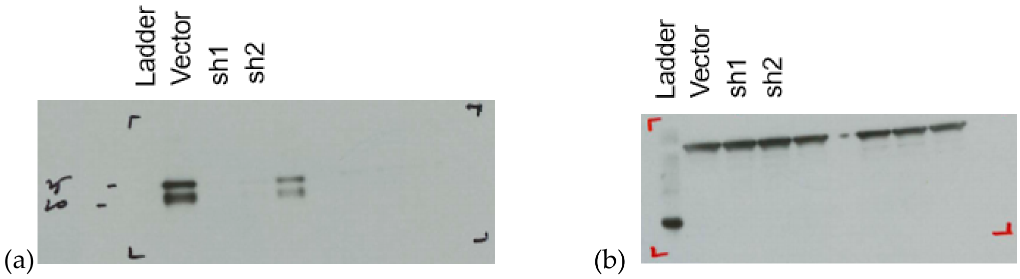

Figure S13. Whole western blot images. **(a)** LY6K western blot. **(b)** GAPDH western blot.

Table S2. Western blot densitometry data. Data given in units.

Sample	GAPDH	LY6K	LY6K/GAPDH
Vector	12110.581	22383.915	1.848
sh1	14239.530	187.850	0.013
sh2	15074.066	268.556	0.018

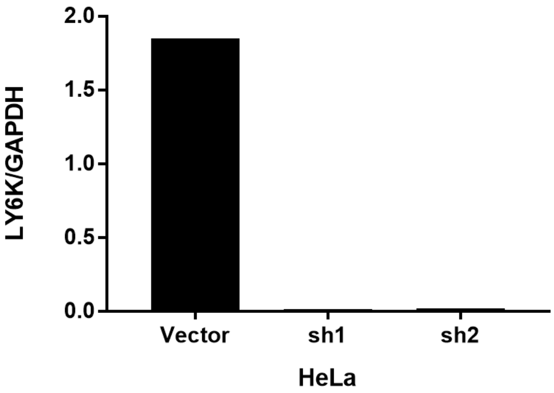

Figure S14. LY6K/GAPDH intensity ratios. Data from Table S2.

© 2020 by the authors. Licensee MDPI, Basel, Switzerland. This article is an open access article distributed under the terms and conditions of the Creative Commons Attribution (CC BY) license (http://creativecommons.org/licenses/by/4.0/).
